# The battle for user-friendly bioinformatics

**DOI:** 10.3389/fgene.2013.00187

**Published:** 2013-09-20

**Authors:** David Roy Smith

**Affiliations:** Department of Biology, University of Western OntarioLondon, ON, Canada

**Keywords:** bioinformatics software, gene annotation, genome assembly, next-generation sequencing, phylogenetics

My first experience of doing scientific research came in the third year of my undergrad when genetics professor Marty Snyder kindly gave me a summer job in her lab at Acadia University. On my first day at work, Marty handed me a CD labeled “scallop data,” and then directed me to a towering Power Mac G4 computer. “See this?” she said, slapping the top of the G4. “Over the next few months you are going to become very well acquainted with this beast.” My assignment was to assemble a segment of the sea scallop genome. To do this, I needed to use a software package called AutoAssembler. “Are you good with computers?” Marty asked. Not really, but I answered yes. “Great,” she said, on her way out the door—“Just insert the CD and click the double-helix icon.” And so began my odyssey into the world of bioinformatics.

AutoAssembler was easy to use, and in no time I was digitally piecing together chunks of scallop DNA. The software had an intuitive, graphical user interface (GUI), which allowed me to drag-and-drop and point-and-click my way to scientific success. For someone who had never done research before, it was exhilarating to see hundreds of DNA sequences and their corresponding chromatograms, like long mountain ranges, spread across the screen. Then poof, with the push of a button, I could transform these genetic puzzle pieces into full-length genes. The experience also gave me the courage to explore other bioinformatics resources online. Before the week was out, I was blasting this, aligning that, and bootstrapping it all together. I was fast becoming a genomic junky, so much so that I asked Marty if I could have a copy of AutoAssembler to use on my laptop computer at home. The answer was no, of course. “Commercial bioinformatics software packages, like AutoAssembler,” Marty explained, “are very expensive and, unfortunately, the lab can only afford one license.” Not to worry, I thought. I'll just download one of the many open-source genome assemblers that are available online. I soon discovered, however, that most of them, although powerful, are command-line driven, can take weeks to learn, and provide little in the way of instruction or technical support. After a few failed attempts at using some of these programs, I scurried back to AutoAssembler with my technological-tail between my legs.

Years later, I found myself on the other side of the country working in a bioinformatics-focused lab where all around me was the buzz of RAM'ed up computers and Linux operating systems, and even the coffee machines seemed like they were command-line driven. In this environment, drag and drop was for amateurs and GUI was a dirty word. But late at night, in the privacy of my one-bedroom apartment, I would covertly run my favorite user-friendly bioinformatics tools. I had CodonCode Aligner for assembling Sanger data, a student license of Geneious for genome annotation and alignments, MEGA for basic phylogenies, and an academic copy of CLC Workbench for next-generation sequence analysis. These programs were more than adequate for addressing most of my bioinformatics needs and were certainly more enjoyable to use than the Unix workstations and barebones programs in the lab. Nevertheless, I did understand why the lab avoided the types of GUI software that I was so fond of: they can be costly, memory-hungry, slow, poor at handling massive datasets, and, because of their complex underlying code, difficult to customize or modify. There is also a lot to be said for mastering the use and theory of the open-source programs upon which the commercial tools are based.

Over time, I discovered that I wasn't the only one in the lab with a penchant for the point and click. Although reluctant to admit to it, my colleagues were impressed by many of the cutting-edge commercial bioinformatics platforms hitting the market, which, unlike their predecessors, were fast, powerful, beautifully designed, and provided wide-ranging functionality. Similar to the operating systems on smartphones, contemporary bioinformatics software suites are multi-faceted, allowing users to download applications (or “plugins”) for specific types of analyses, and integrate both open-source as well as proprietary algorithms, making the software flexible and scalable to users' needs. They also provide an excellent way to organize and access molecular sequence data, and support the import and export of dozens of different file formats. But as one of my lab mates said: “Why should I pay hundreds of dollars for a prettied-up, all-in-one package of programs that I can get for free?” That same person, however, did not think twice about forking out the big bucks on Adobe Photoshop for making publication-quality images.

Free software or not, it seemed like everyone in the department, from ecologists to population geneticists to cell biologists, was dealing with bioinformatics issues. Each day, researchers were stopping by the lab to ask my computer-whiz colleagues for advice. Most had used next-generation sequencing technologies to complement their studies and were looking for straightforward ways to analyze their data. Some had very specific but complex questions, such as, “How do I set up a pipeline for genome assembly and annotation?” Whereas others would ask: “I just received a 5 GB fastq file of Illumina RNA-seq data, what do I do next?” For the latter group, steering them toward easy-to-use software was usually the first and best strategy.

The field of bioinformatics is expanding at an enormous rate and playing an increasingly central role in biological research, medicine, and other diverse facets of human life. From the onslaught of companies specializing in personal genomics, such as *23andMe*, to poets, like Christian Bök, inscribing verse into bacterial genes, soon everyone will be exposed to bioinformatics in one way or another. As new genetic technologies spread to our hospitals, schools, homes and corner stores, we will need equally sophisticated and easy-to-use bioinformatics resources to accompany them. The ability to access and examine molecular sequence data should not be restricted to those with exceptional computer skills; it should be made accessible to all scientists and health practitioners, and the population as a whole.

I recently started my own research lab at a Canadian university, and the first thing I purchased with my startup funds was a computer. The second thing was a user-friendly bioinformatics platform. I hope that these investments will help the undergraduate and graduate students that come through my lab become comfortable with genetic sequence analysis and focus on solving scientific questions rather than computer problems. On one of the shelves in my office is the old laptop computer from my Acadia days; every now and then I boot it up and run the copy of AutoAssembler that Marty eventually bought me.

